# Personalized Medicine of Monoclonal Antibodies in Inflammatory Bowel Disease: Pharmacogenetics, Therapeutic Drug Monitoring, and Beyond

**DOI:** 10.3389/fphar.2020.610806

**Published:** 2021-02-08

**Authors:** Antonello Di Paolo, Giacomo Luci

**Affiliations:** ^1^ Department of Clinical and Experimental Medicine, University of Pisa, Pisa, Italy; ^2^ Unit of Clinical Pharmacology and Pharmacogenetics, Pisa University Hospital, Pisa, Italy

**Keywords:** inflammatory bowel disease, monoclonal antibodies, pharmacokinetics, interindividual variability in drug response, therapeutic drug monitoring, pharmacogenetics

## Abstract

The pharmacotherapy of inflammatory bowel diseases (Crohn’s disease and ulcerative colitis) has experienced significant progress with the advent of monoclonal antibodies (mABs). As therapeutic proteins, mABs display peculiar pharmacokinetic characteristics that differentiate them from chemical drugs, such as aminosalicylates, antimetabolites (i.e., azathioprine, 6-mercaptopurine, and methotrexate), and immunosuppressants (corticosteroids and cyclosporine). However, clinical trials have demonstrated that biologic agents may suffer from a pharmacokinetic variability that could influence the desired clinical outcome, beyond primary resistance phenomena. Therefore, therapeutic drug monitoring (TDM) protocols have been elaborated and applied to adaptation drug doses according to the desired plasma concentrations of mABs. This activity is aimed at maximizing the beneficial effects of mABs while sparing patients from toxicities. However, some aspects of TDM are still under discussion, including time-changing therapeutic ranges, proactive and reactive approaches, the performance and availability of instrumental platforms, the widely varying individual characteristics of patients, the severity of the disease, and the coadministration of immunomodulatory drugs. Facing these issues, personalized medicine in IBD may benefit from a combined approach, made by TDM protocols and pharmacogenetic analyses in a timeline that necessarily considers the frailty of patients, the chronic administration of drugs, and the possible worsening of the disease. Therefore, the present review presents and discusses the activities of TDM protocols using mABs in light of the most recent results, with special attention on the integration of other actions aimed at exploiting the most effective and safe therapeutic effects of drugs prescribed in IBD patients.

## Introduction

The therapy of inflammatory bowel diseases (IBDs), including Crohn's disease (CD) and ulcerative colitis (UC), has been based on aminosalicylates, antimetabolites (i.e., azathioprine, 6-mercaptopurine, and methotrexate), and immunosuppressants (corticosteroids and cyclosporine). These drugs may control symptoms and signs of IBD at the cost of both systemic toxicities and treatment failures observed in a variable percentage of patients (Saibeni et al., 2008; Wahed et al., 2009; Jeong et al., 2019). These issues motivated the scientific community to search for newer pharmacological entities, including monoclonal antibodies (mABs). Thanks to their specific activity against inflammatory processes and their tolerability, mABs represent an area of intense research (Dulai and Sandborn, 2016; Yamamoto-Furusho, 2018; Katsanos et al., 2019).

The clinical use of mABs has shed light on their pharmacokinetic characteristics; a relatively small volume of distribution (approximately equal to plasma and interstitium), a clearance depending on several processes, a negligible renal excretion, and the presence of antidrug antibodies (ADAs) make the pharmacokinetics of mABs of particular interest for interindividual variability, which in turn may depend on genetic polymorphisms. Therefore, the present review will discuss the factors that can affect drug pharmacokinetics, the application of therapeutic drug monitoring (TDM), the role of pharmacogenetic analyses, and their possible integration in the context of personalized medicine for IBD.

## Monoclonal Antibodies Used in Inflammatory Bowel Diseases

The first mABs used in IBD were designed to target the pathway of tumor necrosis factor α (TNFα), which controls cell proliferation and differentiation and promotes a proinflammatory response. Infliximab, adalimumab, golimumab, and certolizumab pegol are prescribed in moderate to severe forms of IBD that respond poorly to other therapies in both induction and maintenance. Indeed, they may ameliorate disease control, reduce hospitalizations and surgery, and finally improve quality of life. Although these are beneficial therapeutic effects, patients may experience a relapse of the disease (Casanova et al., 2017; Bots et al., 2019). The causes behind the failure are not well understood, but individual changes in drug pharmacokinetics and pharmacodynamics or immunogenicity represent possible risk factors. For these reasons, TDM protocols guide dose optimization for every patient on an individual basis.

More recently, mABs can also target extracellular proteins involved in the onset and maintenance of bowel inflammation so it is understood that the number of drugs for the treatment of IBD will increase over the next few years (Hindryckx et al., 2018). In particular, vedolizumab impedes the binding of α4β7-integrin expressed on memory T cells to the mucosal addressin cell adhesion molecule-1 (Mad-CAM-1). The drug is an appropriate therapeutic alternative in IBD patients who developed systemic infections after immunosuppressant regimens or in older patients due to its specific tissue targeting capability for inflammatory processes within gut mucosa (Colombel et al., 2017). Ustekinumab binds the p40 subunit of IL12 and IL23 and blocks the activation of CD4^+^ lymphocytes by activating APCs and their subsequent differentiation into Th1 and Th17 cells (Lamb and Duggan, 2017). As a consequence, the inflammatory cascade through the synthesis and release of several distinct cytokines (i.e., IFNγ, IL2, IL10, IL22, TNFα, and TNFβ) is reduced.

### Therapeutic Monitoring of Monoclonal Antibodies in Inflammatory Bowel Diseases

As presented and discussed in the next sections, many factors may significantly influence and alter the pharmacokinetics of mABs in IBD patients, including possible causes of suboptimal efficacy to treatment or a frank loss of response. TDM protocols may overcome these issues by measuring minimum plasma concentrations (C_min_) and subsequently comparing the values with therapeutic ranges associated with the clinical efficacy of the mABs as defined in clinical trials. In other cases, the therapeutic window of plasma concentrations reflects the improvement in endoscopic endpoints (i.e., mucosal healing) or biomarkers of inflammation, as well as C-reactive protein (CRP) and fecal calprotectin (FCP). In general, the control of the disease in the early phase of therapies (the induction phase) requires higher trough plasma concentrations than in the following postinduction and maintenance phases.

Among the mABs used in IBD, infliximab displays the most extensive collection of results. The suggested lower limit of the therapeutic range of C_min_ values is ≥ 20 mg/L in the induction phase at week 2 (Papamichael et al., 2019a), even if the achievement of mucosal healing in the first weeks of treatment depends on higher plasma concentrations (≥25 mg/L) in both UC and CD patients. That threshold progressively diminishes at week six of therapy (≥10 mg/L, postinduction phase) and, finally it is ≥ 3 mg/L in the postinduction (week 14) and maintenance phases (Vande Casteele et al., 2015), as obtained in CD and UC patients. These trough levels are associated with endoscopic and clinical remission, as well as CRP normalization (≤5 mg/L). However, higher threshold values (≥7 mg/L) at week 14 were associated with clinical remission at weeks 14 and 54 (Kennedy et al., 2019) and mucosal healing (Ungar et al., 2016; Yarur et al., 2017; Papamichael and Cheifetz, 2019). In children, the lower bound of the therapeutic range for trough values of infliximab is 29, 18, and 5.4 mg/L at two, six and ≥14 weeks of treatment, respectively (van Hoeve et al., 2018a, 2018b; Clarkston et al., 2019).

In agreement with these findings, C_min_ values of adalimumab should be higher than 5 mg/L as suggested by several studies (Mazor et al., 2014; Bodini et al., 2016; Nakase et al., 2017), while for mucosal healing and histologic remission, a target range of 8–12 mg/L is recommended (Ungar et al., 2016). Again, C_min_ values of ≥12 mg/L at week 14 predicted clinical remission at both weeks 14 and 54 (Kennedy et al., 2019).

For certolizumab pegol, a former study found C_min_ values > 7.6 mg/L in patients who achieved clinical remission (Colombel et al., 2014), but mucosal healing required higher concentrations (i.e., >19.2 mg/L). More recently, studies identified higher threshold values in the postinduction (>23–36 mg/L) and maintenance phases (>14 mg/L) that were associated with clinical and endoscopic remission and CRP and FCP normalization in CD patients (Vande Casteele et al., 2018; Papamichael and Cheifetz, 2019).

Golimumab needs trough levels >2.5 mg/L at week 6, while a value of ≥1 mg/L is appropriate during maintenance in UC patients (Adedokun et al., 2017). Moreover, trough levels >8.9 mg/L at week 2 (induction phase) predict clinical response (at week 6). In CD patients pretreated with anti-TNFα drugs enrolled in a recent trial, subjects with mucosal healing had golimumab trough levels significantly higher than those who failed to respond (8.9 mg/L vs. 5.08 mg/L, respectively) (Boland et al., 2020); hence, the threshold of golimumab trough concentration was set at 8 mg/L in maintenance therapy.

In the case of vedolizumab, trough values > 28 mg/L at week 2 (and >24 mg/L at week 6) of treatment resulted in clinical response and mucosal healing in the induction phase of UC and CD patients (Williet et al., 2017; Dreesen et al., 2018; Papamichael and Cheifetz, 2019), a threshold value that becomes >20 mg/L in the maintenance phase (week 22) (Dreesen et al., 2018). The probability of clinical remission in both CD and UC patients increased to higher C_min_ values (95%CI, 35–84 mg/L) achieved at week 6 (Rosario et al., 2017). A more recent meta-analysis found a significant association between vedolizumab trough concentrations (>20 and >12 mg/L at week six and at maintenance, respectively) and therapeutic outcomes in UC patients but not in CD individuals (Singh et al., 2019). Of note, some studies questioned the usefulness of vedolizumab TDM (Pouillon et al., 2019) because data are heterogeneous and impede the definition of a clear therapeutic range, while the drug has a low potential for immunogenicity.

Finally, target C_min_ values of ustekinumab decrease from >4 mg/L in the postinduction phase to >0.8 or >1.4 mg/L (depending on the schedule of drug administration) in the maintenance phase (24 and 40 weeks of treatment) (Adedokun et al., 2018). Furthermore, trough concentrations ≥4.5 mg/L were associated with mucosal healing during maintenance (Papamichael and Cheifetz, 2019).

Overall, the concomitant presence of (persistent and rather transient) ADAs can explain the unexpected lower C_min_ values of mABs, as a result of an accelerated clearance. According to proposed algorithms, in the presence of a low ADA titer, patients may continue with the treatment while tailoring the dose to achieve plasma concentrations in the therapeutic range (Derijks et al., 2018). On the contrary, a high ADA titer recommends the prescription of immunomodulatory drugs, the substitution of the current mAB with another, or the switch toward a different drug class (Strik et al., 2017).

It is worth noting that two independent population pharmacokinetic studies found that the titer of ADAs correlated with the estimated clearance values of infliximab (Brandse et al., 2017) and vedolizumab (Okamoto et al., 2020) better than a simple dichotomic presence/absence result. These findings are likely suggesting that a more accurate segmentation of patients according to the ADA titer could further improve the individualization of therapeutic regimens.

### Pharmacokinetics

Table 1 reports the pharmacokinetic characteristics of mABs currently used in the treatment of UC and CD patients (Klotz et al., 2007; Ordás et al., 2012). The molecular weight and structure of mABs influence the passage of drugs across cell membranes during absorption, tissue diffusion, and excretion. The convective transport through a hydrostatic/oncotic gradient between compartments and a sieving effect, which is determined by both the endothelial permeability and the size of mABs, is responsible for transmembrane transport (Ryman and Meibohm, 2017). For example, the diffusion of mABs is higher in the liver and bone marrow (which have sinusoids and fenestrated capillaries, respectively) than that in muscle and skin (characterized by low-permeable capillaries) (Cao et al., 2013). Moreover, in some tissues, reduced convective transport may decrease mAB diffusion (Cooper et al., 2013) so that transcytosis may ensure drug passage through cell membranes and barriers. Transcytosis depends on the binding of mABs to the neonatal Fc receptor (FcRn) expressed on endothelial cell surfaces, and it plays a (minimal) role even in the absorption of mABs after subcutaneous injection (Zhao et al., 2013) thanks to lymphatic vessels. Of note, the presence of an extracellular matrix does delay both the absorption and diffusion of mABs. However, the uptake of drugs by lymphatic vessels depends on lymph flow rate, gradients, and sieving coefficients, resulting in time to peak values between 2 and 8 days for adalimumab, golimumab, and certolizumab pegol, while bioavailability falls in the range 53–80% (Baumann, 2006; Deepak and Loftus, 2016). The loss of mABs during absorption may depend on several factors, as well as degradation within lymph nodes, and cellular uptake mediated by immunoglobulin receptors or due to Fab binding to its target antigen. The latter two mechanisms also account for mAbs excretion together with pinocytosis, as discussed below.

**TABLE 1:** Main pharmacokinetic characteristics of mABs used in UC and CD patients.

	Infliximab	Adalimumab	Golimumab	Certolizumab pegol	Vedolizumab	Ustekinumab
Bioavailability	–	64%^a^	51%^a^ ^,H^	80%^a^	–	57%^a^ ^,CD^
T_max_ (d)	<1 h	5.46 ± 2.3^b^, 8^c^ ^,H^	2–6^d^, 1–7^H^	2.3–7.1	<1 h	7–8.5 ^CD^
Vd (L)	4.5–6.0	4.7–6.0, 7.87^c^ ^,H^	4.1–8.8	7.6	2.73–3.28^e^ ^,H^, 4.84	4.62 ^CD^
T_1/2_ (d)	7.8–13.7^c^	10–20, 16.8^c^ ^,H^	10.9^H^	14	15.1–22^e^, 25.5	19–21 ^CD^
Clearance (ml/h), [L/d]	15.3–18.4, (0.4–10.6)^c^	11–15	20.1 ± 5.8	14.3–19.5	6.5–6.6, (0.136–0.164^e^ ^,H^), (0.159^UC^, 0.155^CD^)	4.6 ^CD^, (0.19) ^CD^
References	Derijks et al. (2018); Hemperly and Vande Casteele (2018); Berends et al. (2019)	Baumann (2006); Sánchez-Hernández et al. (2020), Berends et al. (2019)	Xu et al. (2010); Derijks et al. (2018)	Quetglas et al. (2015); Derijks et al. (2018)	Battat et al., 2019; Berends et al. (2019); EMA (2018)	Lamb and Duggan, 2017

Parameter values are expressed as:

^a^ subcutaneous injection;

^b^ mean ± standard deviation;

^c^ median;

^d^ range;

^e^ mean.

CD, Chron’s disease; UC, ulcerative colitis; H, healthy volunteers.

The mABs bind cell surface receptors or FcγR expressed by many immune cells as well as macrophages, monocytes, NK cells, and neutrophils (Gessner et al., 1998; Hayes et al., 2016). Then, endocytotic internalization brings the drugs within the cytoplasmic endosomes that fuse with lysosomes and mABs are inactivated. The same occurs when the Fab domain binds its target, the so-called target-mediated drug disposition (or TMDD) (Liu, 2018). The TMDD process is saturable at the therapeutic doses of mABs; hence, it does not play a pivotal role in the clearance of these drugs even if it is responsible for a portion of the variable pharmacokinetic profile of biologics. On the contrary, the uptake of mABs by endothelium (the pinocytosis process) seems to have a significant influence on the systemic clearance of mABs because of the larger endothelial surface area in the body, especially in gut, muscle, and skin (Wright et al., 2000).

It is worth noting that the neonatal FcR (FcRn), also known as Brembell receptor, is a salvage pathway for mABs. Indeed, after internalization, FcRn binds IgG and mABs at the acidic pH of the lysosome, so the complex is excluded from proteolysis and directed back to the cell membrane, where the immunoglobulin is newly released in the extracellular space (Israel et al., 1996; Junghans and Anderson, 1996; Liu, 2018). In this manner, the FcRn counteracts the lysosomal degradation of approximately two-thirds of the IgG and mAB (Kim et al., 2007), and the terminal half-lives of therapeutic mABs (10–25 days) are similar to those of endogenous IgG (21 days) in the absence of further confounding factors (Rosario et al., 2015). The only partial exception to this mechanism pertains to certolizumab pegol because it lacks two domains of the constant region (C_H2_ and C_H3_) that bind the receptor. However, the pegylation of certolizumab blocks the glomerular filtration and shields the mAB fragment from the uptake by the reticuloendothelial system (RES). Thanks to these characteristics, certolizumab pegol has a terminal half-life that is comparable with that of other mABs.

The glycosylation pattern of the carbohydrate chains at the Asn297 amino acid within the C_H2_ domain may influence both the pharmacokinetics and pharmacodynamics of mABs (Boune et al., 2020). In particular, mABs characterized by a low content or absence of fucose or sialic acid have shorter terminal half-lives, as well as those harboring a high mannose glycan content. These characteristics partly reflect the effect of glycosylation pattern on FcyR receptor binding and, as a consequence, on drug pharmacodynamics through the enhancement of ADCC and CDC. However, a study achieved different results by using two engineered yeast systems that produced an afucosylated mAB and its variant lacking glycosylation (Liu et al., 2011). Indeed, the *in vitro* binding affinity for FcRn was similar for both mABs and did not differ when compared with that of the same mAB synthesized in CHO cells. Interestingly, when injected in transgenic mice harboring the human FcRn, mABs produced in yeast systems shared the same pharmacokinetic characteristics. Overall, those findings suggested that glycosylation seemed incapable of influencing mAB-FcRn binding at least in those models.

The immunogenicity of the humanized or human IgG1-like mABs used for IBD is still present, and the production of ADAs is another cause of accelerated clearance. For this reason, TDM protocols consider both the drugs and the corresponding ADAs (see below).

Finally, the high molecular weight excludes mABs from filtration, but renal glomeruli can filter minor fragments, which are reabsorbed and metabolized in the extracellular space surrounding the proximal tubule (Waldmann et al., 1972).

The last note regards the distribution of mABs. As mentioned above, the physicochemical characteristics of the drugs, the endothelium permeability, and the presence of extracellular matrix proteins may affect the tissue diffusion of mABs. Overall, the volume of distribution (Vd) is in the range 8–20 L, thus approaching the extracellular body water (Dirks and Meibohm, 2010). Of note, Vd of vedolizumab and ustekinumab ranges between 4 and 5 L (Rosario et al., 2015; Deepak and Loftus, 2016).

### Pharmacokinetic Variability

Several studies and population pharmacokinetic models evaluated the causes of variability between and within IBD patients to identify subgroups at higher risk of treatment failure and consequently candidates for treatment optimization (Table 2). Of note, some of those factors are changing over time (due to progressively better control of the inflammatory process), and modeling is carefully considering this characteristic (Vande Casteele et al., 2017). However, further factors may explain part of that pharmacokinetic variability as in the case of genetic variants of FcRn or the genetic background of patients. Therefore, a mixed pharmacokinetic and pharmacogenetic approach would be more attractive.

**TABLE 2:** Factors significantly associated with changes in mABs clearance, increase ([+]) or decrease ([–]), according to findings from population pharmacokinetic studies.

Covariates	Infliximab	Adalimumab	Golimumab	Certolizumab pegol	Vedolizumab	Ustekinumab
Serum albumin	(−)^a^		(−)^a^	(−)	(−)	(−)
ADA	(+)^b^	(+)	(+)^a^	(+)	(+)^b^	(+)
Immunomodulators	(−)		(−)MTX			
Body weight	(−)^a^/(+)WT	(+) (WT or BMI or LBW)	(+)	(+) (BSA)	(+)	(+)
Inflammation markers		FCP(+)	CRP [+]	CRP (+)	CRP (+), FCP(+)	
Other	Sex (−)^F^, ESR (+)^c^	UDASC, PEN	ALK (+)		Age (+), previous anti-TNFα (+)	Sex (+)^M^
References	Brandse et al. (2017); Hemperly and Vande Casteele (2018); Santacana et al. (2020); Bauman et al. (2020)	Sharma et al. (2015); Vande Casteele et al. (2019); Sánchez-Hernández et al. (2020)	Xu et al. (2018); Berends et al. (2019); Dreesen et al. (2020); Adedokun et al. (2020)	Wade et al. (2015)	Rosario et al. (2015); Osterman et al. (2019); Okamoto et al. (2020)	Xu et al. (2020)

Abbreviations: ADA, antidrug antibodies; ALK, alkaline phosphatase; BMI, body mass index; BSA; body surface area; CRP, C-reactive protein; ESR; erythrocyte sedimentation rate; FCP, fecal calprotectin; LBW, lean body weight; MTX, methotrexate; PEN, pen device (40 or 80 mg); UDASC, unexplained decline in adalimumab serum concentrations.

Female (^F^) or male (^M^) patients;

^a^ also in pediatric patients;

^b^ plasma ADA titration;

^c^ only in pediatric patients.

#### Absorption and Distribution

The absorption through the subcutaneous route may be influenced by changes in subcutaneous composition, blood perfusion, and lymph flow rate (Gibney et al., 2010). For example, UC patients weighing >100 kg or ≤100 kg had the same exposure after administration of golimumab at doses of 90 mg or 45 mg, respectively (Sandborn et al., 2008, Sandborn et al., 2012). Moreover, multivariate analysis identified BMI as a factor influencing adalimumab PK in a dose-escalation study (Bultman et al., 2012). The difference in doses did not correspond to a similar well-defined change in body weight, meaning that a single factor may only explain part of the interindividual variability. Indeed, as anticipated above, the administration of mAB at therapeutic doses may saturate the FcRn-dependent passage of the mAB from the injection site into the lymphatic vessels (McDonald et al., 2010) and the subsequent diffusion from blood to tissues.

From a purely pharmacokinetic point of view, body weight remains the most important factor associated with the variability in Vd (Rosario et al., 2015). Indeed, population pharmacokinetic studies identified body weight as the covariate exerting a significant effect on distribution of mABs used in IBD in both adult patients (Hemperly and Vande Casteele, 2018; Adedokun et al., 2020; Xu et al., 2020) and children (Sharma et al., 2015; Xu et al., 2018; Bauman et al., 2020). Sex may be an additional significant factor for Vd (Ternant et al., 2008; Fasanmade et al., 2009) as well as previous treatments with anti-TNFα mABs (Dreesen et al., 2020) and race and body surface area (which recalls patient’s body weight) (Wade et al., 2015).

#### Elimination

As anticipated above, the elimination of mABs depends on the interplay of different mechanisms, and many of them are related to the severity of inflammatory status.

The increased concentration of endogenous IgG in severe, active disease may have opposite effects on mABs pharmacokinetics. Indeed, high concentrations of endogenous IgG may compete with mABs to bind with both FcRn (Morell et al., 1970) and FcyR, resulting in a shortened half-life of mABs or a rise in their plasma concentrations. Moreover, the binding of mABs to FcRn is species-specific so that further variability is expected when comparing different mABs.

The FcRn gene harbors a polymorphism consisting of variable number tandem repeats (VNTRs), with a decreased receptor expression associated with the VTNR2 allele at the cellular level. In turn, this leads to a diminished systemic exposure (as the area under the curve) of both infliximab (−14%) and adalimumab (−24%) in heterozygous VNTR3/2 IBD patients in comparison to VNTR3/3 homozygotes during induction (Billiet et al., 2016). These results suggest that FcRn has an effect on mAB absorption from subcutis.

Interestingly, higher serum concentrations of albumin correlated with reduced infliximab clearance (Fasanmade et al., 2009) and with increased systemic exposure to the drug (Fasanmade et al., 2010) (Table 2). Moreover, increased clearance of vedolizumab and ustekinumab corresponded with reduced values of serum albumin (Rosario et al., 2015; Lamb and Duggan, 2017). The reason of these relationships could be the competition for FcRn binding during the elimination process or the higher loss of proteins (including mABs) in the presence of the most severe inflammatory status.

Other markers of disease severity and inflammation may predict changes in mAB pharmacokinetics. Elevated serum concentrations of CRP were associated with increased clearance of both certolizumab pegol (Wade et al., 2015) and golimumab (Adedokun et al., 2020) and with lower plasma concentrations of ustekinumab (Lamb and Duggan, 2017). FCP correlated with the increased clearance of adalimumab (Sánchez-Hernández et al., 2020) and vedolizumab (Osterman et al., 2019), while erythrocyte sedimentation rate significantly influenced the clearance of infliximab in children (Bauman et al., 2020) (Table 2). Finally, a study reported the passage of infliximab into the gut lumen through the inflamed mucosa by an unknown mechanism (Brandse et al., 2015), as demonstrated in patients affected by the most severe UC (Kapel et al., 1992).

ADA in patients’ plasma may be associated with poor treatment efficacy, while their production may be variable among the anti-TNFα agents, ranging from ≤2.3% for ustekinumab (Deepak and Loftus, 2016; Feagan et al., 2016), up to 25.3% for infliximab (Thomas et al., 2015), with intermediate percentage values for adalimumab (14.1%), certolizumab (6.9%), and golimumab (3.8%). Furthermore, the concomitant administration of other drugs, such as azathioprine and methotrexate, may modulate the production of ADA. Indeed, the infliximab-AZA combination was associated with a reduced incidence of ADAs (0.9% *vs*. 14.6%), increased C_min_ values of mAB, and a higher rate of corticosteroid-free remission rate than the sole infliximab (Seow et al., 2010). The combinations infliximab-thiopurines and adalimumab-methotrexate brought ADAs to undetectable levels in 77% of patients who previously experienced a loss of response due to immunogenicity (Strik et al., 2017). Even in children, methotrexate significantly reduced the clearance of infliximab (Xu et al., 2018) (Table 2), likely reducing the ADA production.

Of note, the formation of ADAs may depend on the schedule of the mAB regimen. Indeed, the occurrence rate of ADAs is higher after an occasional administration of mABs rather than a regular regimen (Colombel et al., 2010; Khanna et al., 2013). Moreover, the genotype at the HLA-DQA1*05 locus predicts the magnitude of the immune response against infliximab or adalimumab (Sazonovs et al., 2020). This finding may help in defining a combined signature to screen IBD patients who are candidates to receive mABs as discussed below.

Finally, other factors can induce changes in mAB pharmacokinetics (Table 2). For example, vedolizumab clearance increased with body weight values higher than 120 kg (Rosario et al., 2015). Body weight also affects ustekinumab clearance, for which the sex and race of patients were additional factors (Lamb and Duggan, 2017). However, the relationship between infliximab clearance and body weight may vary over the induction and maintenance phases. In particular, non‐obese patients could be underdosed during the induction phase comparing to obese patients (Dotan et al., 2014), but the latter tend to clear infliximab more rapidly during maintenance, as it was observed with adalimumab (Billiet et al., 2016).

## Personalized Medicine in Inflammatory Bowel Diseases

**FIGURE 1:**
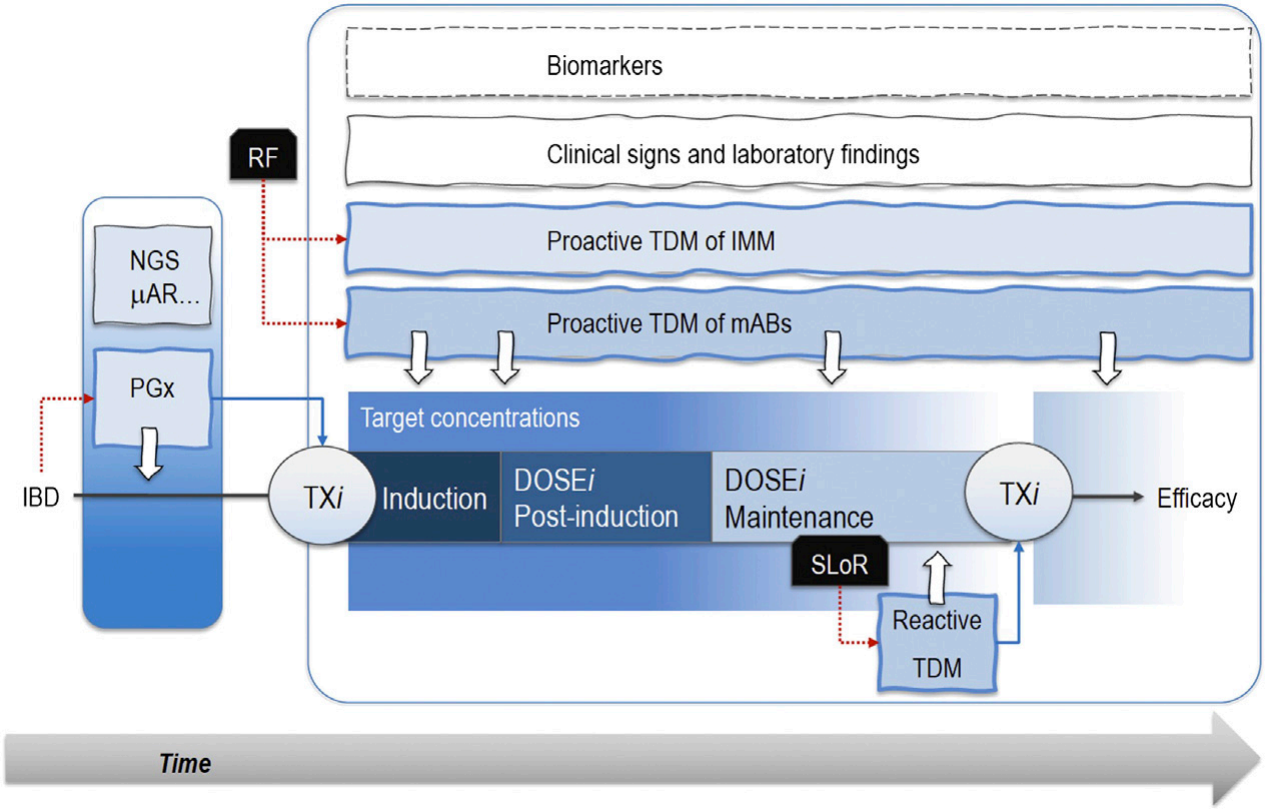
Pharmacogenetic analyses (PGx) through next-generation sequencing (NGS) or microarrays (μAR) allow patients’ segmentation and personalized pharmacological therapies (TXi) early in the induction phase. Notably, known risk factors (RF) associated with treatment failure sustain proactive therapeutic drug monitoring (TDM) protocols based on the measurement of drug plasma levels. Therefore, patients receive optimized drug doses (DOSE*i*) in the post-induction and maintenance phases when proactive TDM still mantains its role. The occurrence of a secondary loss of response (SLoR) triggers a reactive TDM that may support the decision about a possible change in drug regimen. These approaches take advantage of TDM of immunomodulatory drugs (IMM), clinical signs, laboratory findings, and biomarkers (i.e., C-reactive protein, fecal calprotectin, TNFα serum concentrations). Dotted red lines are the actions emerged from a critical event (i.e., the IBD diagnosis or SLoR), while the dotted blue lines represent the following therapeutic choices. White arrows are the analyses for PGX or TDM protocols..

Both drug monitoring protocols and pharmacogenetic analyses are valuable in exploiting the therapeutic effect of mABs while sparing patients from toxicities. Of note, these endpoints may be combined with clinical markers of efficacy and tolerability (i.e., age, disease severity, and extension) or markers of inflammation (FCP and CRP) to increase the predictive performance of the phenotypic and/or genetic signature (Figure 1).

### Therapeutic Drug Monitoring

TDM protocols for mABs significantly improve the management of IBD patients in comparison with empiric dose changes based on signs, symptoms, and laboratory results. Low minimum plasma concentrations suggest a dose increase, whereas values within the therapeutic range in association with reduced clinical response indicate a change of drug is needed, as it occurs in the presence of high ADA concentrations (Bendtzen et al., 2009). Furthermore, the optimization of mABs dose does not require a short turnaround time of the test because the long half-life (10–25 days) allows for blood collections two or three days before the next injection, a time that is compatible with the routine execution of the test.

In IBD patients, the therapeutic range of each mAB depends on the severity of the disease and on the endpoint chosen to guide therapy (i.e., clinical or endoscopic remission or normalization of some laboratory findings). It is worth noting that some points deserve a discussion: (1) homogeneity of therapeutic ranges; (2) proactive and reactive TDM; (3) the appropriateness of TMD; (4) the most suitable instrumental platform to quantitate serum concentrations of mABs; (5) the turnaround time of TDM, and finally (6) the strategies that combine TDM of mABs, immunomodulatory drugs, and pharmacogenetic analyses.

#### Therapeutic Ranges

The identification of a therapeutic range for a drug takes place in clinical trials aimed at establishing a relationship between the administered dose, plasma concentrations, clinical response, and toxic effects. In IBD, the severity of the disease may affect this relationship because a suboptimal clinical or endoscopic/histologic outcome could be related to the most severe disease rather than lowest trough values. Furthermore, therapeutic ranges should reflect the different phases of treatment (induction, postinduction, and maintenance phase) for better control of the active disease in the first phase. A mild albeit therapeutic effect is required in the maintenance phase to keep the disease under pharmacological control for the longest time in the absence of toxic effects.

It is worth noting that the lower bounds of therapeutic ranges also reflect the population considered. As reported above, the recommended trough plasma values of infliximab are higher in children compared with adult patients. Indeed, in 35 children with IBD (23 with CD and 12 with UC), high infliximab C_min_ values (median 6.0 mg/L, range 3.2–12.0 mg/L) during maintenance correlated with combined clinical/biological remission at week 52, whereas low trough concentrations did not (median 2.6 mg/L, range 1.1–3.2 mg/L) (van Hoeve et al., 2019). More recently, another study demonstrated that the percentage of patients with subtherapeutic trough levels (i.e., <5.4 mg/L) at week 14 was higher in young children (<10 years old) than in older ones (≥10 years old) (Jongsma et al., 2020). Importantly, underdosed children required dose increases and developed ADA more frequently than older patients.

Finally, the studies may adopt different endpoints, which include plasma threshold values for clinical remission, mucosal healing, histologic/endoscopic remission, normalization of laboratory exams, and biomarkers (FCP). All of these considerations should also take into account a possible variability between the instrumental platforms and the manufactured tests used for the measurement of mABs and ADA concentrations in plasma (as discussed below). Therefore, many factors can influence the identification of a target therapeutic range, lastly including patient’s compliance (Gibson et al., 2020).

#### Reactive and Proactive Therapeutic Drug Monitoring

Overall, reactive (Papamichael and Cheifetz, 2016) and proactive (Mitrev et al., 2017) approaches for mAB TDM are aimed at comparing drug concentrations and the eventual presence of ADAs to target therapeutic ranges and guide dose optimization. The striking difference between the two is that reactive TDM is suitable when patients under therapy have an unsatisfactorily clinical response, and it can be helpful in the management of secondary loss of response. On the contrary, the proactive approach is appropriate for patients with risk factors for treatment failure (for example, most severe disease and previous anti-TNFα therapies) or most severe consequences after the loss of response (i.e., need for surgery).

The proactive approach has some advantages compared with reactive protocols (Papamichael et al., 2019c) because it is associated with better outcomes, as demonstrated for infliximab (Papamichael et al., 2018) and adalimumab (Papamichael et al., 2019b). Notably, a study found that infliximab trough levels decreased during the first year of treatment; hence, the researchers suggested that “close monitoring of the IFX-TLs (trough levels) could be recommended during maintenance IFX treatment even for patients in remission to be more alert and act a priori” (Orfanoudaki et al., 2019). Furthermore, a prospective Australian study evaluated the impact of TDM on the prescription of infliximab in a real-world setting (Wu et al., 2019), and it found that TDM helped to identify avoidable infliximab dosing with a cost saving. Interestingly, the inappropriate administration was more frequent in reactive (38.9%) than in proactive TDM (19.3%). Data regarding TDM in pediatric studies are scarce (Aardoom et al., 2019), but the adoption of a proactive TDM could be helpful also in children, for whom “IBD is extensive, safety is paramount, and experience with newer biologics is limited” (Carman et al., 2018). For these reasons, proactive TDM is gaining more attention to keep disease under pharmacological control, to prevent loss of response, and to avoid toxicities (Penagini et al., 2020; Vermeire et al., 2020). Interestingly, a third study demonstrated that the incidence of infliximab discontinuation, drug trough levels, and the presence of ADA did not differ between the proactive TDM and the empiric dosing when associated with immunomodulation (Lega et al., 2019).

On the other hand, two systematic reviews and meta‐analyses compared empiric dose adjustment and reactive and proactive TDM (Ricciuto et al., 2018; Shah et al., 2020). The first study failed to demonstrate a full advantage for TDM in comparison to the empiric approach in terms of remission rates (Ricciuto et al., 2018). The only significant improvement resided in a cost-saving effect for reactive TDM and a long therapeutic benefit to anti-TNFα drugs for proactive TDM. In partial agreement with these results, the second study found that only proactive TDM gave some advantages against both the empiric approach (RR, 0.60, 95%CI 0.35–1.04) and reactive TDM (RR, 0.22, 95%CI 0.15–0.22). However, as stated by the authors, the analyses included studies that were heterogeneous for many characteristics, with “very low quality of evidence, mainly due to risk of bias, inconsistency, and imprecision” (Shah et al., 2020).

Overall, TDM is superior to empiric dose adjustment for anti-TNFα drugs, and the reactive approach is a standard of care for IBD patients. More recently, some data are suggesting a propensity of proactive TDM to exploit all of the therapeutic benefits of anti-TNFα mABs (see next section), but the proactive approach is not unanimously considered a standard of care.

#### Timing of Monoclonal Antibody Therapeutic Drug Monitoring

A panel of experts recently agreed on the timing of TDM for anti-TNFα drugs in IBD patients (Papamichael et al., 2019a). Indeed, proactive TDM is appropriate at the end of the induction phase and at least once in the maintenance period. Patients with primary suboptimal or lack of response and with secondary loss of response would receive reactive TDM (as already discussed in the previous section). For other biologic agents used in IBD (i.e., vedolizumab and ustekinumab), TDM may be considered appropriate at the end of the induction phase (proactive) and when a secondary loss of response is demonstrated (reactive) despite the fact that evidence concerning these drugs is limited (Papamichael et al., 2019a).

#### Instrumental Platform for Monoclonal Antibody Therapeutic Drug Monitoring

The immunoassays (i.e., ELISA) represent the laboratory reference methods for mAB TDM; thanks to their compatibility with widely diffused platforms already in use in clinical biochemistry laboratories. These characteristics ensure that the majority of IBD patients could benefit from TDM protocols. Different immunoassays for the measurement of plasma concentrations of infliximab and its corresponding ADAs were interchangeable because the findings were significantly correlated (Marini et al., 2017; Nasser et al., 2018), hence strengthening the reliability of TDM from the perspectives of patients and caregivers. Furthermore, based on the available evidence, the variability among tests for infliximab and its ADAs was not clinically significant while the tests did not return different results for infliximab and its biosimilars (Papamichael et al., 2019a). The last finding deserves attention because of the broader use of infliximab biosimilars, even in children (Jongsma et al., 2017).

Although the demonstrated performance of immunoassays, systematic bias, suboptimal specificity, and lack of standardization may characterize the different ELISA methods (Schmitz et al., 2016; van Bezooijen et al., 2016) with inconsistent threshold values of mABs across the studies. The sensitivity and drug tolerance of the available ADA assays represent additional concerns (Egging et al., 2018; Nice et al., 2020). For these reasons, researchers have been engaged in the elaboration and validation of TDM protocols based on liquid chromatography coupled with mass spectrometry (LC-MS). The development of such methods is sometimes laborious (An et al., 2014), the most problematic step being the preparation of peptides by enzymatic digestion (Mouchahoir and Schiel, 2018). Indeed, validated methods often require high expertise in sample preparation and instrumental analysis (Chiu et al., 2018; Willeman et al., 2019), characteristics that may impede the adoption of methods among laboratories.

However, the validation of LC-MS methods for infliximab, adalimumab (Jourdil et al., 2018; El Amrani et al., 2019), and vedolizumab (Schulze et al., 2018) returned findings significantly correlated with those obtained with ELISA immunoassays. Interestingly, the application of an LC-MS method to infliximab TDM gave a plasma concentration threshold of 6.2 mg/L for biological remission (plasma CRP<5 mg/L and FCP<150 μg/g stools) of the disease (Nemoz et al., 2019), a value that was in the lower band of the therapeutic range suggested for mucosal healing in the maintenance period (Ungar et al., 2016; Yarur et al., 2017). In another trial, an LC-MS method did monitor trough levels of vedolizumab in IBD patients (Schulze et al., 2018). Responders had mean values (38.3 and 41.8 mg/L) higher than those of patients with loss of response (33.4 and 39.3 mg/L) in the induction period (weeks two and six of treatment, respectively).

#### Therapeutic Drug Monitoring of Thiopurines

As stated above, the pharmacological management of IBD may require the administration of thiopurines. However, a correlation may exist between combined treatments and an increased risk for infections and neoplasms (Bots et al., 2018). A solution to reduce those unfavourable outcomes is to administer immunomodulatory drugs for a limited time and under TDM supervision when possible. Indeed, 6-TGN levels >105–125 pmol/8x10^8^ erythrocytes were required to reduce infliximab immunogenicity and, hence, ADA production (Colombel et al., 2010; Yarur et al., 2015). Similar findings were obtained for adalimumab when 6-TGN concentrations were ≥223 pmol/8x10^8^ erythrocytes (sensitivity 100% and specificity 60.6%) (Nakase et al., 2017). In the case of vedolizumab, concomitant immunosuppressive therapy decreased the formation of ADAs (Feagan et al., 2013; Sandborn et al., 2013). On the contrary, immunomodulation did not affect ustekinumab clearance (Lamb and Duggan, 2017). Therefore, although the modulating effect of thiopurines could be generalized in all patients, their use in combination with mABs may be considered on a case-by-case basis due to the risk of increased adverse reactions.

### Pharmacogenes in Inflammatory Bowel Diseases

The evaluation of genetic markers associated with treatment efficacy or tolerability is relevant for both chemical and biologic drugs.

In 2014, Bank and coworkers found that 19 out of 39 functional polymorphisms in 26 genes belonging to the NFκB-mediate inflammatory response predicted the response to anti-TNFα therapy in a large cohort of 482 CD and 256 UC patients (Bank et al., 2014). In 2016, a meta-analysis found that polymorphisms on TLR2, TLR4, TLR9, TNFRSF1A, IFNγ, IL6, and IL1β genes correlated with treatment response in IBD (Bek et al., 2016). Additionally, another polymorphism on the FCGR3A gene was predictive of therapy response in CD patients.

Notably, in 2014, Bank and colleagues replicated the gene signature in a subsequent cohort of 587 CD and 458 UC patients (Bank et al., 2019). The updated signature included ten polymorphisms belonging to NFκB-, TNFα-, and cytokine-signalling pathways. Interestingly, patients with risk signatures for TNFα-driven inflammatory status were most likely prone to experience a benefit from anti-TNFα agents. A subsequent study partially confirmed those findings in 103 IBD patients (80 CD and 23 UC) (Romero-Cara et al., 2018). In particular, a correlation emerged between the wild-type allele of the polymorphism rs396991 (V158F) in the *FCGR3A* gene and the development of ADAs (highest plasma concentration in VV homozygous patients) and reduced concentrations of infliximab. Overall, the studies well stressed the need to investigate multiple causative factors to obtain a genetic signature with good predictive/prognostic performance.

The genetic analyses may pair with clinical or laboratory findings. For example, in 29 CD and 18 UC patients, a study detected a significant correlation between the polymorphism rs1143634 within the promoter region of the IL1β gene, the higher cytokine concentrations at baseline, and reduced response (in terms of clinical remission) to infliximab at week 14 (Lacruz-Guzmán et al., 2013). Similarly, the polymorphism rs2228273 in ZNF133, with thiopurine use and body weight, predicted unsatisfactory response to infliximab after the first administration (Jung et al., 2019).

In recent years, pharmacogenetic analyses have taken advantage of the employ of unsupervised techniques, as well as microarrays, GWAS, and next-generation sequencing platforms, which enable the screening of numerous possible genetic markers at the same time (Di Paolo et al., 2019). In some cases, genetic markers were combined with clinical and laboratory characteristics to obtain highly performant predictive models (Dubinsky et al., 2010). For example, in 231 UC patients of Caucasian ancestry, a recent study found two gene signatures of 8 and 12 SNPs associated with primary non-response (PNR) and duration of response (DR) to anti-TNFα therapies, respectively (Burke et al., 2018). Intriguingly, “genetic risk scores for PNR and DR were not associated with infliximab levels or antibody formation” meaning that “the associations of these SNPs may be mediated by mechanisms other than drug pharmacokinetics or antibody formation.” In UC patients, another study evaluated whether a genetic signature developed for infliximab could also predict mucosal healing, clinical response, and remission after treatment with golimumab (Telesco et al., 2018). The findings demonstrated that the signature was mainly drug-specific because it could not identify patients who did achieve clinical remission or response after golimumab treatment. The study also evaluated a possible companion diagnostic for anti-TNFα agents (Kaneider and Kaser, 2018). In 474 IBD patients of European ancestry, two genetic variants, rs116724455 in TNFS4/18 and rs2228416 in PLIN2, were predictive of refractoriness to therapy and increased the predictability of a clinical-based risk model (Wang et al., 2019). Finally, a Spanish research group found a significant association between five polymorphisms in TNFα or NFκB pathways and plasma concentrations of both infliximab (rs5030728 in TLR4 and rs11465996 in LY96) and adalimumab (rs1816702 in TLR2, rs2569190 in CD14, and rs3397 in TNFRS1B) in 154 children affected by IBD (Salvador-Martín et al., 2020) although different regimens and the number of patients in subgroups partly weakened those associations.

The genetic signature discovered by GWAS may be partially disease-specific, as in the study by Jostins et al. (Jostins et al., 2012) who revised 15 GWASs and found that 110 IBD loci were shared between CD and UC, whereas 30 and 23 loci were specific for CD and UC, respectively.

Finally, a very recent GWAS performed in 1,240 biologic-naïve patients found a significant association between the HLA-DQA1*05 locus and both an increased rate of immunogenicity (OR, 1.90) and the development of ADAs against infliximab and adalimumab (Sazonovs et al., 2020). Of note, the use of the biologics alone or in combination with other drugs did not affect this relationship. In another study that enrolled 252 IBD patients, the variant allele of the HLA-DQA1*05 locus significantly increased the risk for ADAs against infliximab (HR = 7.29) independently from age, sex, weight, and immunomodulators (Wilson et al., 2020), which are known factors affecting the elimination of mABs.

## Discussion

Tailoring pharmacological therapy for every IBD patient means choosing an effective therapeutic regimen that could be promptly modified in the presence of poor responsiveness to the disease or after the onset of toxic effects. Additional reasons may sustain treatment optimization on an individual basis, as well as the chronic administration of drugs even in combination, their low therapeutic index, and the progressive worsening of the disease. According to these factors, four sequential steps may increase the therapeutic potential of mABs by exploiting their pharmacodynamic and pharmacokinetic characteristics (Figure 1).

First of all, pharmacogenomic tests may score the patient’s risk of unsatisfactory response to mABs at the time of diagnosis, well before the patient will be considered a candidate to receive a biologic agent (Wang et al., 2019). Indeed, germinal genetic variants may be predictive biomarkers of response to mABs, as done to define the risk of developing CD or UC in different populations (Venkataraman and Rivas, 2019). It is worth noting that in many cases the prediction can be improved by a genetic signature rather than using a single genetic locus.

This strategy may also evaluate the risk of disappointing efficacy or toxicities associated with thiopurines, methotrexate, aminosalicylates, and immunosuppressants (Voskuil et al., 2019), both in adults (Heap et al., 2014; Heap et al.,2016; Kakuta et al., 2018; Walker et al., 2019) and children (Lucafò et al., 2019). This approach is valuable when TDM protocols require chromatographic methods that can be laborious and need specific expertise as in the case of thiopurines. Moreover, pharmacogenomic analyses may predict the tolerability of combined treatment regimens, which are advantageous as second-line therapies (Roblin et al., 2020). For example, a Dutch study did define a genetic passport that included several loci (TPMT, NUDT15, HLA-DQA1*02:01-HLA-DRB1*07:01, and HLA-DQA1*05) associated with toxic effects from thiopurines (i.e., myelosuppression and pancreatitis) and anti-TNFα mABs (i.e., immunogenicity) (Bangma et al., 2020). The signature may work even in combination with clinical risk factors (i.e., previous anti-TNFα therapies) (Rosario et al., 2017), assuming that it is cost-effective in terms of both additional costs for healthcare systems and patients’ quality of life (Sluiter et al., 2019). However, the presence of rare variant alleles could weaken that relationship (Zimdahl Kahlin et al., 2019), hence justifying “the use of a combination of genotyping and phenotyping in order to detect as many individuals as possible who are at risk of treatment failures and adverse reactions during thiopurine treatment”.

According to that suggestion, the second step considers proactive TDM to better define the responsiveness status during or at the end of the induction phase for responders and nonresponders to anti-TNFα agents, as well as for nonresponders to vedolizumab and ustekinumab (Papamichael et al., 2019a). This approach is mainly valuable for carriers of known risk factors, and this is advantageous in comparison with following empiric dose adjustment or reactive TDM based on signs and symptoms of the disease (Ricciuto et al., 2018; Shah et al., 2020). Although characterized by some drawbacks, immunoassays represent the methods with the broadest diffusion across laboratories.

The third step involves TDM during the maintenance phase when the measurement of mABs and ADAs may be appropriate for all anti-TNFα agents (Papamichael et al., 2019a), while the reactive TDM can be the standard of care for all IBD patients who experienced a loss of response. As presented in previous paragraphs, the titration of ADAs (rather than the notification of their presence or absence) could further improve the segmentation of patients according to their likelihood of response (Brandse et al., 2017; Okamoto et al., 2020).

It is worth noting that the TDM protocols in the second and third steps may take advantage of specific proactive activities, as well as dose optimization according to algorithms that may represent the fourth step. Indeed, *in silico* studies demonstrated that adaptive dosing strategies were superior to stepwise or proportional dosing approaches (Wojciechowski et al., 2017) and two clinical trials evaluated Bayes models (Strik et al., 2019; Santacana Juncosa et al., 2020). In particular, the work of Santacana Juncosa and colleagues demonstrated that the regimen individualization according to a Bayes strategy may significantly increase the percentage of patients in clinical remission and the remission rate for those who need an intensified dosage. Moreover, the disease control lasted in the deintensified cohort, and 92.4% of patients still received infliximab after one year of treatment (Santacana Juncosa et al., 2020).

In conclusion, a combined pharmacokinetic/pharmacogenetic approach may achieve new goals in the treatment of IBD patients; thanks to a tailored approach based on TDM (including Bayes strategies), pharmacogenomic analyses, and clinical records. Likely, adding pharmacodynamic markers (i.e., plasma, cellular, or tissue levels of cytokines as TNFα and IL8) may increase the predictive value of models and, ultimately, the control of the disease with significant improvements in patients’ health status and quality of life.
